# Reliability of panoramic ultrasound imaging and agreement with magnetic resonance imaging for the assessment of lumbar multifidus anatomical cross-sectional area

**DOI:** 10.1038/s41598-023-46987-z

**Published:** 2023-11-10

**Authors:** Daniel P. Fitze, Martino V. Franchi, Loris Peterhans, Walter O. Frey, Jörg Spörri

**Affiliations:** 1 Sports Medical Research Group, Department of Orthopaedics, Balgrist University Hospital, University of Zurich, Zurich, Switzerland; 2 University Centre for Prevention and Sports Medicine, Department of Orthopaedics, Balgrist University Hospital, University of Zurich, Zurich, Switzerland; 3 Institute of Physiology, Department of Biomedical Sciences, University of Padua, Padua, Italy

**Keywords:** Skeletal muscle, Ultrasound

## Abstract

The aim of this study was to investigate the reliability of panoramic ultrasound (US) imaging and agreement with magnetic resonance imaging (MRI) for assessing the average lumbar multifidus anatomical cross-sectional area between the lumbar vertebral bodies L3–L5 (i.e., LMF ACSA_L3–L5_). US and MRI scans of 20 male youth competitive alpine skiers were collected. To test the intra- and interrater reliability of US, transversal panoramic scans were analyzed on two different days by the same rater and the analysis of the first day was compared with the analysis of a second rater. To examine the agreement between US and MRI, Bland–Altman analysis was performed. Intrarater reliability was *excellent,* and interrater reliability was *weak to good* for both sides. The bias between MRI and US was − 0.19 ± 0.90 cm^2^ (2.68 ± 12.30%) for the left side and − 0.04 ± 0.98 cm^2^ (− 1.11 ± 12.93%) for the right side (i.e., for both sides US slightly overestimated LMF ACSA_L3–L5_ on average). The limits of agreement were − 1.95 to 1.57 cm^2^ (− 26.70 to 21.30%) for the left side and − 1.95 to 1.88 cm^2^ (− 26.46 to 24.24%) for the right side. Panoramic US imaging may be considered a method with *excellent* intrarater and *weak to good* interrater reliability for assessing LMF ACSA_L3–L5_. Comparison with MRI showed large individual differences in some cases, but an acceptable bias between the two imaging modalities.

## Introduction

The lumbar multifidus (LMF) anatomical cross-sectional area (ACSA) appears to play a crucial role in the context of low back pain^1–3^. Several studies using different imaging modalities, such as magnetic resonance imaging (MRI)^4–6^ and ultrasound (US) imaging^7–9^, have found differences in LMF ACSA between symptomatic and asymptomatic patients with low back pain. For example, a retrospective study^4^ found that 80% of 78 patients with low back pain experienced LMF muscle atrophy. In addition, a large-scale study^5^ of 412 adult participants showed that fat infiltration in the LMF was strongly associated with low back pain in adults. Finally, a prospective study^6^ found selective ipsilateral atrophy of the LMF in unilateral low back pain specific to the symptomatic side in 50 low back pain patients. Furthermore, it seems that differences between symptomatic and asymptomatic low back pain are mainly localized in the lower lumbar region from lumbar vertebral bodies L3 to L5^7,9^. Studies investigating the effect of loading or unloading found that hypertrophy or atrophy was primarily confined to this region^10–12^. Thus, quantifying changes in LMF ACSA in individuals with low back pain could provide valuable information that may be used for tailored therapeutic interventions^9^.

While MRI represents the gold standard for obtaining muscle ACSA^13^, transversal panoramic US imaging is a reliable and valid alternative to measure ACSA^14,15^. Since MRI is costly and its availability is limited, the advantages of US are that it can be portable and cost-effective^16^. In the context of LMF, several studies have shown that US imaging is a reliable method to quantify ACSA^17–22^. However, studies comparing US- and MRI-based values are scarce, and those that have compared imaging modalities for LMF ACSA have not used panoramic US.

For example, Hides and colleagues^23^ found no significant differences between the two imaging modalities when comparing the LMF ACSA between L2-S1 in young asymptomatic individuals. Sions and colleagues^24^ also concluded that US is a valid alternative to MRI, even in older symptomatic and asymptomatic patients, for measuring L4 LMF ACSA. Conversely, Belavý and colleagues^25^ found only a *poor to moderate* correlation between the US and MRI measurements. To the best of our knowledge, however, we are not aware of any studies that have compared transversal panoramic US images (a feature that is available only on newer US systems) with MRI images. Such an approach becomes necessary as soon as the muscle ACSA to be measured is wider than the field of view of the transducer. Another advantage of panoramic imaging is the fact that the left and right side of the LMF ACSA can be displayed in the same scan, which would otherwise only be possible with a convex transducer. Accordingly, the aim of this study was to investigate the intra- and interrater reliability of the panoramic US protocol and to compare the values of US and MRI in the clinically relevant muscle region L3–L5.

## Methods

### Study design and participants

In this cross-sectional study, the data from 20 male youth competitive alpine skiers (age = 15.01 ± 0.45 years, height = 1.68 ± 0.09 m and body mass 55.30 ± 10.20 kg) were analyzed. The underlying study protocol was approved by the local ethics committee of the Canton of Zurich (KEK-ZH-NR: 2017-01395) and was conducted according to the ethical standards of the Declaration of Helsinki and national laws. All participants provided written informed consent.

### Ultrasound imaging

**Figure 1:**
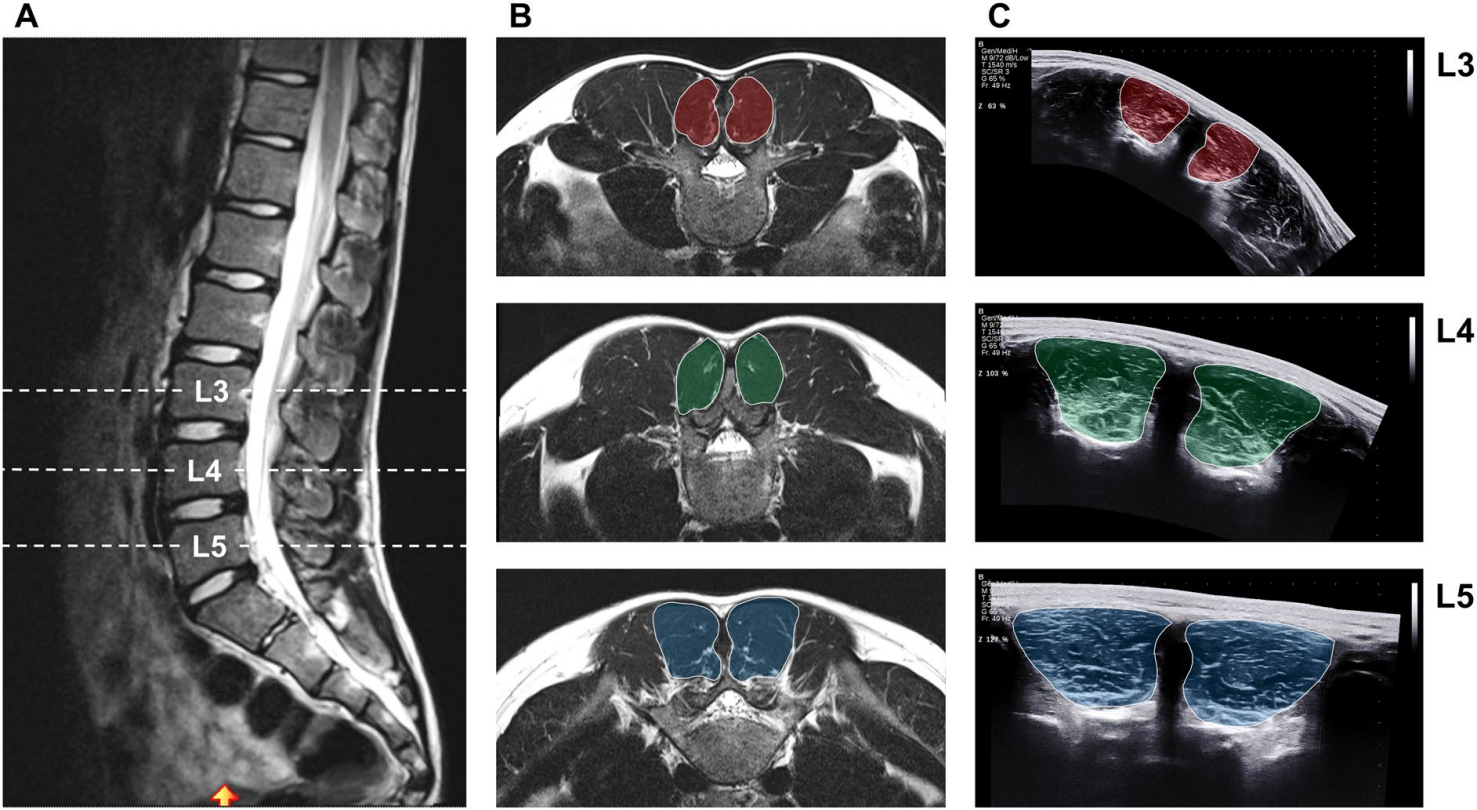
Representative MRI and US images of the LMF ACSA at lumbar vertebral bodies L3-L5. (**A**) Sagittal MRI image with identified lumbar vertebral bodies L3-L5; (**B**) Transversal MRI images of LMF ACSA at L3, L4 and L5; (**C**) Transversal panoramic US images of LMF ACSA at L3, L4 and L5. *MRI* magnetic resonance imaging, *US* ultrasound, *LMF ACSA* lumbar multifidus anatomical cross-sectional area.

Participants were positioned in a prone position while keeping their ankles on the edge of the bed so that their feet could be maintained in a neutral position and their hip and knee joints were extended. A pillow was additionally placed under each participant's abdomen to ensure a neutral position of the lumbar spine. US acquisitions were carried out by an expert operator (MVF) (i.e., with more than 10 years of experience with muscle US) with an ultrasound system (Aixplorer Ultimate, SuperSonic Imagine, Aix-en-Provence, France) using a 4-cm linear transducer (SuperLinear SL10-2, SuperSonic Imagine, Aix-en-Provence, France). The image depth was adjusted individually according to the participant and the scan frequency varied depending on the image depth. First, the lumbar vertebral body levels L3, L4 and L5 were identified by a longitudinal US scan and marked on the skin (on both sides). The time required for identification and marking of the L3–L5 measurement planes (usually between 3 and 5 min) served to restore natural fluid redistribution. This duration was considered sufficient because the participants had previously been lying in the MRI for 1 h and only had to change the examination room for the US measurement. Subsequently, transversal panoramic US images were acquired for the marked lumbar vertebral body levels L3-L5 (Fig. 1C) (always in the following order: L5, L4, L3). For this purpose, a generous amount of ultrasound gel was applied to the imaging pathway, and the transducer was swept slowly over the region of interest with constant pressure, always starting from the right side towards the left side. One US scan was performed for each region.

Analysis of the images was performed via image processing software (ImageJ, National Institutes of Health, Bethesda, MD) by an experienced rater (DPF, rater 1) (i.e., with more than 5 years of experience in manual segmentation of muscle US images). For this purpose, the images were first scaled, and if necessary, the brightness and contrast were adjusted so that the boundaries of the LMF were visible as well as possible. Subsequently, the ACSAs of the right and left side were traced and measured in randomized order (i.e., sometimes starting with the right and sometimes with the left side) using the polygon selection tool. The manual segmentation of the US images was repeated one day later by the same rater to calculate the intrarater reliability. In addition, a second rater (LP, rater 2) analyzed the US images in the same manner once for the calculation of interrater reliability.

### Magnetic resonance imaging

Before the US measurement, all participants underwent an MRI examination of the lumbar spine in a supine position. A 3 Tesla MRI scanner (Magnetom Prisma, Siemens, Erlangen, Germany) with a specific spine coil (32-channel receiver) was used to perform this procedure. The scan protocol involved an axial T2-weighted turbo spin-echo sequence (repetition time (TR), 5430 ms; echo time (TE), 96 ms; slice thickness, 4 mm; field of view (FOV), 320 mm × 20 mm; matrix, 384 × 384). All examinations were performed by a radiographer. For image analysis, the central segments of the lumbar vertebral bodies L3-L5 were identified in the sagittal plane (Fig. 1A), and the ACSA was measured in the associated transversal plane (Fig. 1B). LMF ACSA analysis was obtained once by rater 1 through image processing software (ImageJ, National Institutes of Health, Bethesda, MD) following the same approach as for the US images.

### Statistical analysis

For statistical analysis, US and MRI values were averaged between L3 and L5 per side. When interpreting the data, it is therefore important to be aware that the data represent an average value from a total of three analyzed slices per side (i.e., ACSA_L3–L5_). The data were tested for normal distribution using the Shapiro–Wilk test, which was given in all cases, through statistical software (SPSS Statistics 26, IBM, Armonk, USA). To determine the intra- and interrater reliability, the spreadsheet for consecutive pairwise analysis by Hopkins^26^ was used. The spreadsheet was used to calculate intraclass correlation coefficients ICC(3,1). ICC confidence intervals (CIs) were classified based on the definitions of Koo and Li^27^: < 0.5 indicates *weak* reliability, 0.5 to 0.75 indicates *moderate* reliability, 0.75 to 0.9 indicates *good* reliability, and > 0.9 indicates *excellent* reliability. In addition to the ICC values, the standard error of measurements (SEMs) and the minimal detectable changes (MDCs) were calculated. SEMs were calculated as Ref.^28^: $SEM=SD \,of \,change \times \sqrt{1-ICC}$ and MDCs were calculated as Ref.^29^: $MDC=1.96 \times \sqrt{2} \times SEM$. To examine the agreement between US and MRI, Bland–Altman analyses^30,31^ were performed for each side with GraphPad Prism 9.0.0 statistical software (Insight Partners, New York, United States). The absolute and relative differences of the two imaging modalities (i.e., the first US analysis from rater 1 with the MRI analysis from the same rater) were plotted as a function of the mean values, and the resulting average differences (biases) were illustrated. In addition, the upper limits of agreement (ULOAs), and the lower limits of agreement (LLOAs) were determined (± 1.96 SD) and plotted.

## Results

### Intra- and interrater reliability of ultrasound imaging

Table 1 shows the results of the intra- and interrater reliability analysis. The intrarater reliability for LMF ACSA_L3–L5_ of the left side was found to be *excellent* (ICC(3,1): 0.98, 95% CI 0.95–0.99). The SEM and MDC were 0.04 cm^2^ and 0.11 cm^2^, respectively, for this site. The right side also showed *excellent* intrarater reliability (ICC(3,1): 0.98, 95% CI 0.96–0.99). For this site, the SEM and MDC were 0.04 cm^2^ and 0.11 cm^2^, respectively. The interrater reliability for LMF ACSA_L3–L5_ of the left side was found to be *weak to good* (ICC(3,1): 0.75, 95% CI 0.47–0.89). The SEM and MDC were 0.45 cm^2^ and 1.25 cm^2^, respectively, for this site. The right side also showed weak to *good* interrater reliability (ICC(3,1): 0.66, 95% CI 0.36–0.85). For this site, the SEM and MDC were 0.58 cm^2^ and 1.61 cm^2^, respectively.

**Table 1:** Intra- and interrater reliability for assessing the average lumbar multifidus anatomical cross-sectional area between the lumbar vertebral bodies L3–L5 (i.e., LMF ACSA_L3–L5_).

	ICC (3,1)	95% CI	SEM (cm^2^)	MDC (cm^2^)
Intrarater reliability				
LMF ACSA_L3–L5_ left	0.98	0.95–0.99	0.04	0.11
LMF ACSA_L3–L5_ right	0.98	0.96–0.99	0.04	0.11
Interrater reliability				
LMF ACSA_L3–L5_ left	0.75	0.47–0.89	0.45	1.25
LMF ACSA_L3–L5_ right	0.66	0.32–0.85	0.58	1.61

*LMF* lumbar multifidus, *ACSA* anatomical cross-sectional area, *L3-L5* average between the lumbar vertebral bodies L3-L5, *ICC* intraclass correlation coefficient, *95% CI* 95% confidence interval, *SEM* standard error of measurement, *MDC* minimal detectable change.

### Comparison of ultrasound and magnetic resonance imaging

**Figure 2:**
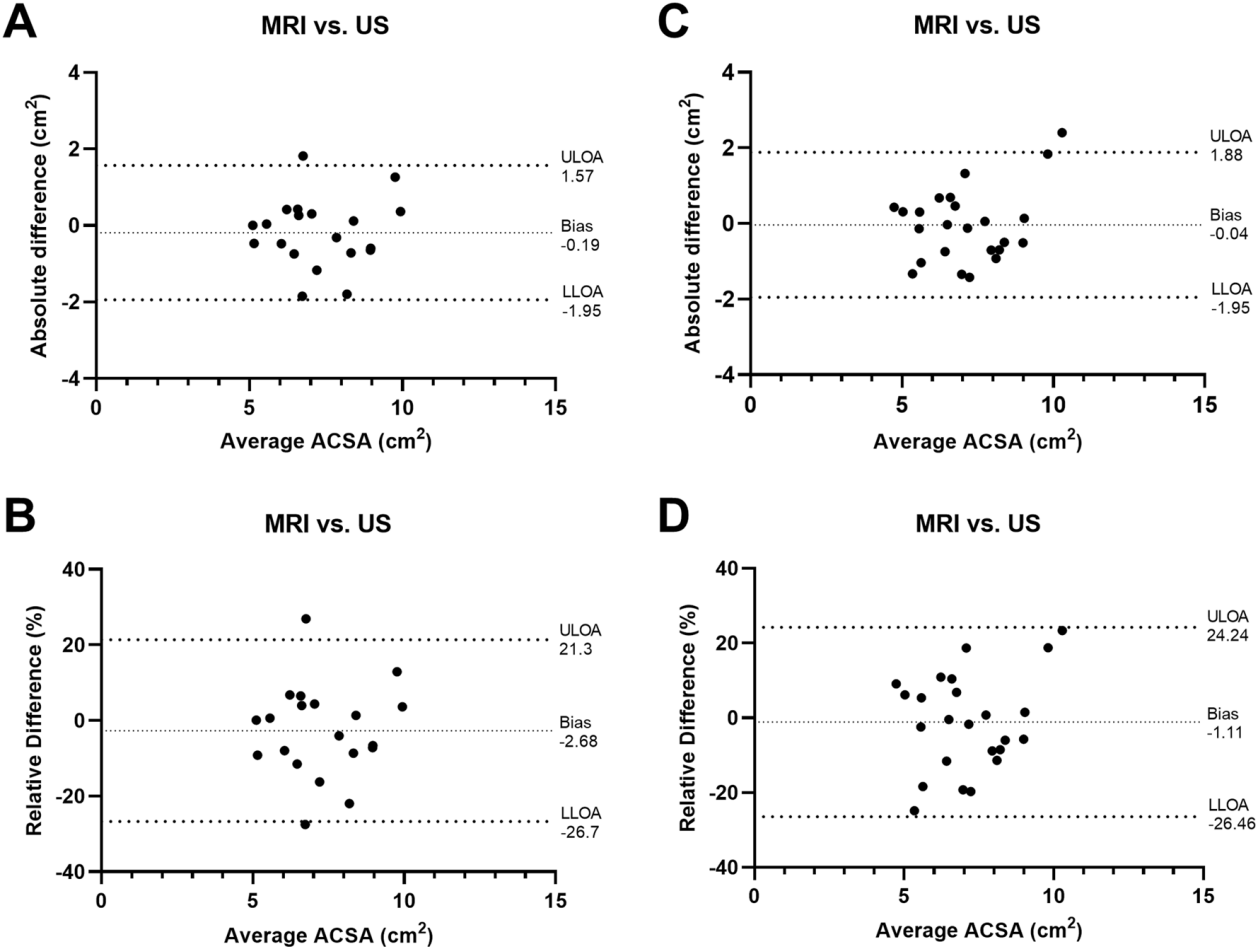
Bland–Altman analysis of the comparison of MRI vs. US for assessing LMF ACSA_L3-L5_. (**A**) Absolute bias, LLOA and ULOA for the left side; (**B**) Relative bias, LLOA and ULOA for the left side; (**C**) Absolute bias, LLOA and ULOA for the right side; (**D**) Relative bias, LLOA and ULOA for the right side. *MRI* magnetic resonance imaging, *US* ultrasound, *LMF ACSA*_*L3-L5*_ lumbar multifidus anatomical cross-sectional area averaged for the lumbar vertebral bodies L3-L5, *Bias* average difference, *LLOA* lower limit of agreement, *ULOA* upper limit of agreement.

Figure 2 shows the results of the Bland–Altman analysis. The absolute bias between the two measurement methods for the left side was − 0.19 ± 0.90 cm^2^, the LLOA was -1.95 cm^2^ and the ULOA was 1.57 cm^2^. This corresponded to a relative bias of − 2.68 ± 12.30%, an LLOA of − 26.70% and a ULOA of 21.30%. For the right side, the absolute bias was − 0.04 ± 0.98 cm^2^, the LLOA was − 1.95 cm^2^ and the ULOA was 1.88 cm^2^. This corresponded to a relative bias of − 1.11 ± 12.93%, an LLOA of − 26.46% and a ULOA of 24.24%.

## Discussion

The major findings of the present study were as follows: (1) panoramic US can be considered an imaging modality with *excellent* intrarater reliability and *weak to good* interrater reliability for measuring LMF ACSA_L3–L5_; (2) the comparison between panoramic US and MRI revealed acceptable average differences (less than 3% for both sides), but in some cases relatively high individual differences (up to 27%).

The ICC(3,1) CIs (left side: 0.95–0.99, right side: 0.96–0.99), the SEMs (left and right side: 0.04 cm^2^) and the MDCs (left and right side: 0.11 cm^2^) from the intrarater reliability analysis in the present study were comparable to those from Wilson and colleagues^18^. They found ICC(3,1) CIs (L3: 0.93–0.98, L4: 0.88–0.96, L5: 0.92–0.98), SEMs (L3: 0.20 cm^2^, L4: 0.37 cm^2^, L5: 0.37 cm^2^) and MDCs (L3: 0.56 cm^2^, L4: 1.03 cm^2^, L5: 1.03 cm^2^). Thus, although the present study produced slightly higher values for the ICC(3,1) CIs and slightly lower values for the SEMs and MDCs, it must be considered that these resulted from the average ASCA of L3–L5 (i.e., LMF ACSA_L3–L5_) and not from individual lumbar vertebral levels. Previous studies^19–22^, which repeated both the US measurement and the analysis with the same operator/rater to analyze the intrarater reliability (i.e., test–retest reliability), also showed slightly lower values for the ICC(3,1) CIs and slightly higher values for SEMs and MDCs. This can be explained by the fact that the present study was exclusively focused on the intra- and interrater reliability of the US analysis. Nonetheless, one of the possible reasons for the *excellent* intrarater reliability could be the high image resolution capacity of the US system utilized in the present study, which leads to the generation of images with high quality and thus allows for a better recognition of the LMF boundaries. A further explanation may be that the subjects in the present study were youth competitive alpine skiers, which is a sample where muscle atrophy and the associated fat infiltration are not typically prevalent^5^. Nonetheless, recent studies with youth competitive alpine skiers from our group showed that a smaller relative LMF ACSA was significantly associated with the more frequent occurrence of overuse-related spinal abnormalities^32^ and that asymptomatic subjects showed greater LMF ACSA at L5 than symptomatic subjects with overuse-related back complaints^33^. Given these findings, it is reasonable to assume that this is a sample in which the measurement of LMF ACSA may be of relevance in the prevention of low back pain.

For interrater reliability, we found only *weak to good* reliability for both sides. In comparison to the study from Wilson and colleagues^18^, where the authors assessed the interrater reliability for LMF ACSA L5, the values from the present study were smaller for ICC(3,1) CIs and larger for SEMs and MDCs. This suggests that manual segmentation of panoramic US images for LMF ACSA was more rater dependent in our study. Possible reasons for this result include that the LMF boundary from the erector spinae is often unclear, the two muscles have a similar grey distribution to the background, and the shape of paraspinal muscles can be very individual^34^. Potentially, the differences in the backgrounds and experience of the two raters also led to different interpretations of the LMF's boundaries.

When the US and MRI images were analyzed by the same rater (rater 1), the Bland–Altman analysis revealed a relative bias of less than 3% for both sides, whereas the LLOA and ULOA reached values of up to 27% and 25%, respectively. These values are comparable to the results of the study by Belavý and colleagues^25^. The found negative bias for the left and right sides further indicated that US overestimated the LMF ACSA_L3-L5_ on average. A contributing factor for this result could be the different lying positions between the two imaging modalities. While the skiers were lying supine during MRI, they were lying prone during the US measurement. Theoretically, due to compression in the supine position during MRI, the ACSA could be artificially reduced^24^. Another potential influencing factor could be the difficulty of standardizing the measurement position and the image plane between the two imaging modalities. While the identification of the lumbar vertebral bodies L3-L5 is relatively straightforward with MRI in the sagittal plane, this proves to be much more difficult via longitudinal scan using US. Furthermore, the orientation of the transducer during the scan can have a great influence on the ACSA of the muscle to be measured.

The present study has some limitations. Firstly, the sample consisted solely of a limited number of youth male competitive alpine skiers, which may limit the transferability of the study results to other samples and settings. Secondly, while the identification of the lumbar vertebral bodies is relatively straightforward with MRI in the sagittal plane, this proved to be more difficult with a longitudinal US scan. Thus, potentially not exactly the same positions and planes may have been compared between US and MRI.

## Conclusion

Panoramic US imaging can be considered a method with *excellent* reliability if the same rater analyses the images on different days and a method with *weak to good* reliability if different raters analyze the same images for the assessment LMF ACSA_L3–L5_. This suggests that manual segmentation of panoramic US images was to some degree rater dependent. The relative biases of less than 3%, but values of up to 27% and 25% for the lower and upper limits of agreement from the US and MRI comparison showed that although there was an acceptable average difference, there can be very high individual differences between the two imaging modalities.

## Data Availability

The datasets generated and/or analyzed during the current study are not publicly available but are available from J.S. (joerg.spoerri@balgrist.ch) on reasonable request.
